# Do Infants at Risk of Developing Cerebral Palsy or Other Neurodevelopmental Disorders Learn What They Practice?

**DOI:** 10.3390/jcm9072041

**Published:** 2020-06-29

**Authors:** Kristina Löwing, Linda Holmström, Rita Almeida, Ann-Christin Eliasson

**Affiliations:** 1 Neuropediatric Unit, Department of Women’s and Children’s Health, Karolinska Institutet, SE-171 76 Stockholm, Sweden; kristina.lowing@ki.se (K.L.); linda.holmstrom@ki.se (L.H.); 2 Stockholm University Brain Imaging Center (SUBIC), Stockholm University, SE-106 91 Stockholm, Sweden; rita.almeida@su.se

**Keywords:** early intervention, cerebral palsy, development, other neurological disorder, gross motor function, upper limb function

## Abstract

Through secondary analyses of the Small Step. Randomized Control Trial, we tested the hypothesis that children at risk of developing cerebral palsy (CP) or other neurodevelopmental disorders would learn what they practice, i.e., that they would have a more rapid development within the specifically trained foci (hand use or mobility) of each time period compared to the development rate within the foci not trained at that time. Nineteen infants (6.3 (1.62) months corrected age) included in the Small Step program were assessed at six time points during the intervention. For statistical analysis, general and mixed linear models were used, and the independent variables were the Peabody Developmental Motor scale (stationary, locomotion, grasping and visuomotor sub scales), the Gross Motor Function Measure-66 and the Hand Assessment for Infants. Outcomes related to gross motor function improved significantly more after mobility training than after hand use training, while fine motor function was improved to the same extent following both training types. Significantly higher improvements after the first training period were seen in one out of three outcome measures in both gross and fine motor assessments. The improvements observed were all independent of diagnosis at two years. The concept “you learn what you practice” was most clearly confirmed in the case of gross motor development.

## 1. Introduction

Early interventions designed for children at risk for cerebral palsy (CP) or other neurodevelopmental disorders exploit the activity-dependent plasticity and rapid development of the central nervous system during the first years of life. Neurobiological research has suggested that the effectiveness of neural networks and pathways can be strengthened by early intervention, and the first thousand days after birth have been identified as the most critical period of development [1,2,3,4]. However, a recent systematic review concluded that early intervention programs exert only weak positive effects on the outcome of infants at risk of developing CP [5]. At the same time, similar interventions have had a distinct positive impact in the case of toddlers and pre-school-aged children with CP [6,7,8,9,10]. To make use of the theoretical potential of early intervention and improve efficacy, also in the youngest children, we need to develop interventions specific to this age group and learn more about how to best design the content and structure of intervention. 

The current investigation extends the findings of a recent Randomized Controlled Trial (RCT) [11] that evaluated the effects of the Small Step Program on young children. This program is based on principles of motor learning, including a combination of incorporated intervention elements to provide individualized, goal-directed, and self-initiated activity during an intensive period of training that addresses different foci (mobility, hand use, and communication) within specific time-frames, implemented in the child’s home environment by the their parents (who have been coached by therapists) [12,13]. Similar interventional elements have previously been suggested to be included in other early intervention programs [14,15]. In the Small Step Program, the children are randomly assigned to first train either hand use or mobility and thereafter train the other foci. The underlying concept is that each skill should be trained specifically and repeatedly in order to selectively strengthen the involved nerve pathways, i.e., that you should practice what you want to learn [16,17,18].

The design of the Small Step Program allows us to explore how the temporal organization and the different elements of the intervention contribute to outcome. Our hypothesis was that children at risk of developing CP or other neurodevelopmental disorders would learn what they practice, i.e., they would have a more rapid development within the specifically trained foci of each time period compared to the development rate within the foci not trained at that particular time. Other research questions in this study were whether it mattered for the overall outcome if the infants started with mobility or hand use, as well as whether there was any difference in improvement rate between the different time periods in the Small Step program.

## 2. Materials and Methods

**Figure 1:**
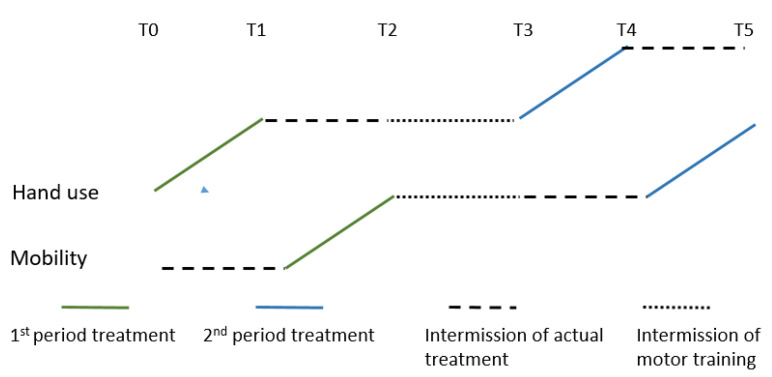
Schematic illustration of the study design. Children were assigned randomly to begin training either their hand use or mobility. This figure is an example that shows when children begin with hand use and thereafter receive mobility training. After these two periods, there is an intermission without motor training. Thereafter, the second block of training starts. T0–T5 comprises the different time points for assessments.

A secondary analysis of the previously published RCT including the 19 children belonging to the intervention group in the Small Step Program was performed [11]. In the RCT, the children were randomly assigned to first train mobility or hand use. As shown in Figure 1, data were collected at six different time-points (T0–T5) separated by approximately 7 weeks, the intervention lasting for a total of about 35–40 weeks. The T2–T3 period consisted of communication training. Results from this period are not presented here, so this period is referred to as the intermission of motor training in this paper. The therapists who collected the data were not involved in the intervention and were blinded with respect to previous assessments of the children. Further details are presented elsewhere [12].

The data were collected from 2014 to 2018 at the Astrid Lindgren Children’s Hospital, a tertiary hospital in Stockholm, Sweden. This study was pre-approved by the Stockholm Regional Ethical Review Board (no. 2013/2044-31/1).

### 2.1. Participants

The demographic characteristics, functional ability, and diagnoses of the 19 infants included are presented in Table 1. At two years of age, 9 children had been diagnosed with CP, 8 with other neurodevelopmental disorders, and 2 with no diagnosis. Epilepsy and extensive comorbidity were common (*n* = 5). It should also be noted that at two years of age, those with neurodevelopmental disorders other than CP all scored below the age-normative score on all subscales of the Peabody Developmental Motor scale (PDMS-2) except for the grasping subscale. Almost all children who were not diagnosed with CP at two years were classified at level I on the Gross Motor Function Classification System—Expanded and Revised (GMFCS—E&R) and Mini-Manual Ability Classification system (MACS) classifications. For further information on the recruitment procedure, see [11].

### 2.2. Brief Description of the Intervention

The Small Step Program included three different foci (mobility, hand use, and communication), the first two of which are analyzed here. The rationale for dividing the intervention program into hand use, mobility, and communication was to help the families to optimize the training within each foci of training. Training was conducted daily and, as mentioned earlier, in the home, by the parents, who received weekly coaching and supervision from either physiotherapists or occupational therapists responsible for the specific foci (a more detailed description is provided in the study protocol) [12].

### 2.3. Brief Description of the Different Foci of Training

The training of hand use is based on the assumption that effective grasping helps an infant to plan ahead. The development of manual dexterity and different aspects of hand–eye coordination are essential aspects of cognitive development in infants. Exciting toys, chosen carefully to be suitable to each infant’s present level of hand function, were used to stimulate self-initiated actions. For the youngest children and/or those with the lowest ability, actions as simple as reaching out to touch their mother’s nose could be meaningful; meanwhile, as the child’s awareness and fine motor development improve, the task had to become more complex. With all children, it was necessary to continuously vary objects in order to maintain attention and to optimize the conditions for development (for a more detailed description of hand use, see [12].

The training of mobility is based on the assumption that children have an inner drive to move and explore their environment. The objective here was to optimize the infant’s possibilities to move and perform daily life as they grow older by increasing postural control and mobility. This training was also individualized on the basis of each child’s special strengths and interests. Less “hands on” assistance was essential and, by learning through trial and error, the children took pleasure in the increasing competence of their own bodies [19]. Their parents were also encouraged to use everyday situations to promote training, such as placing the child in front of a parent’s drawer to motivate the child to stand up and investigate the different objects. For a more detailed description of mobility, see [12].

### 2.4. Outcome Measures

Four subtests of the PDMS-2 (0–5 years) were used [20]. Two subtests—the stationary (score 0–60) and the locomotion subscales (0–130 score)—were related to mobility, and two subtests—the grasping (0–52 score) and visual motor integration (0–144 score) subscales—were related to hand use.

The Gross Motor Function Measure-66 (GMFM-66), an observational criteria-referenced measure was used to evaluate changes in the gross motor function of the children. The items are organized in order of increasing difficulty, from 0 (low capacity) to 100 (high capacity). GMFM-66 has shown good psychometric properties [21,22,23]. The Hand Assessment for Infants (HAI) was used, another observational and criteria-referenced measure with excellent interrater and test retest reliability that assesses hand use in 3–12-month-old infants at risk of developing CP on a scale from 0 (low capacity) to 100 (high capacity) [24,25]. The Hammersmith Infant Neurological Examination (HINE) and Alberta Infant Motor Scale (AIMS), both assessments that have good psychometric properties, [26,27,28,29] were used here to control for potential differences in baseline characteristics. Bayley Scales of Infant Development (BSID-III) cognitive index and diagnoses was administered at 2 years of age.

### 2.5. Statistical Analysis

Analysis was carried out using the R-program (version 3.2.3, Vienna, Austria). Descriptive statistics of frequencies, means, SD, and confidence intervals were used to describe the data. After confirming that the distribution of values was normal according to the Shapiro–Wilk test, the Wilcoxon signed-rank test or paired t-tests were used to compare the baseline (T0) and T5 for the 6 outcome measures of interest. The different time periods (T0–T5) are further described in Figure 1.

In addition, the data were examined utilizing linear or, wherever possible in the case of repeated measurements of an outcome for the same child, linear–mixed models. In the latter case, the subject was considered to be a random variable. The scores for the six items on the GMFM-66, the stationary and locomotor subscales of the PDMS-2 and HAI, and the grasping and visuomotor subscales of PDMS-2 at T5, as well as changes in these variables between subsequent measurements, were the outcome (dependent) variables. 

The potential influence of the order of training, i.e., hand use or mobility first, on the final outcome (i.e., at T5) was examined using a linear model, with the starting foci and the various outcome measure at baseline as independent variables.

To analyze whether the first or second period of training exerted the greater impact, the change scores in outcome after each period were used as dependent variables, while the order of training periods and the HINE at baseline were the independent variables. 

To examine whether the specific foci of training (hand use or mobility) affected the outcome after the period of corresponding training, the changes in outcome (combined for both time periods of hand use and mobility training) were treated as the dependent variables, while the scores on the different scales representing type of training (mobility or hand use) and the HINE at baseline were used as independent variables. The same procedure was used for the type of training versus intermission of motor training. We also tested separate models including the interaction between the HINE at baseline and foci of training. The interactions were never significant, and the results of those models are thus not further discussed. We also performed these same comparisons against the AIMS values at baseline, GMFCS—E&R, BSID-III cognitive index, and a diagnosis of CP.

The GMFCS—E&R levels were divided into two different groups, one including levels I–II and the other including levels III–V (GMFCS—E&R/2). Diagnosis was also analyzed in a binary fashion, i.e., as the presence or absence of a diagnosis of CP. 

## 3. Results

### 3.1. Do Children Improve With Training?

**Figure 2:**
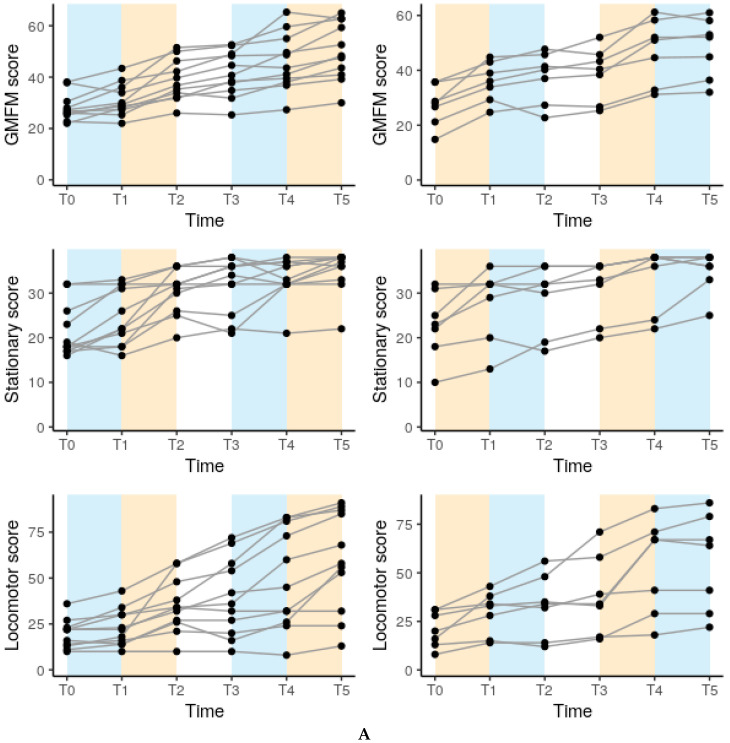

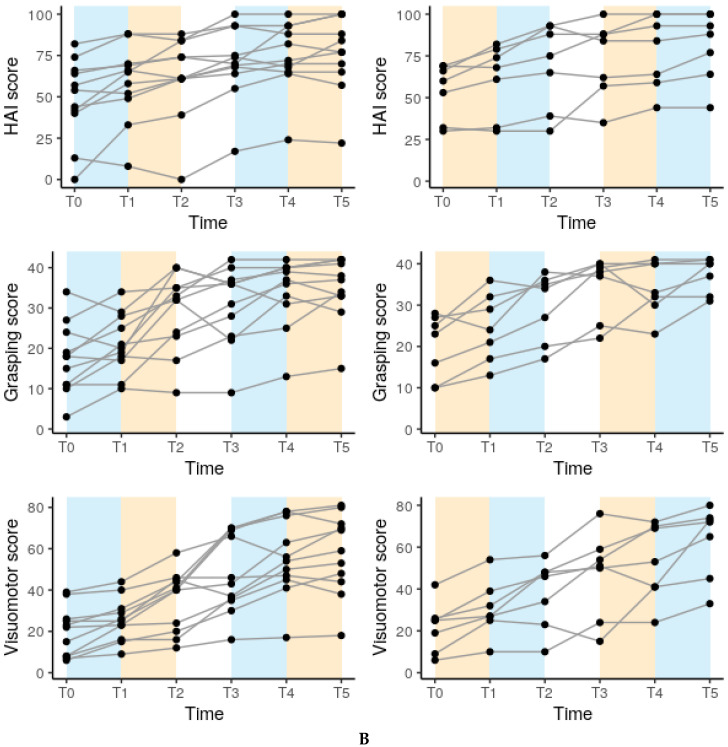
Individual trajectories of the different outcomes over the course of the intervention. Hand use periods are indicated in blue, mobility training periods in orange, and intermission of motor training in white. The plots on the left show the trajectories of infants that started with hand use training, and the plots on the right show the trajectories of those who started with mobility training. (**A**) Infants starting with mobility training. (**B**) Infants starting with hand use training. For further clarification of the time points T0–T5, see Figure 1.

The children’s scores for all six outcomes (GMFM-66 and the stationary, locomotor, grasping, and visuomotor subscales of the PDMS-2 and the HAI) were significantly improved after the intervention (*p* < 0.0003 for all) (shown in Table 2). Figure 2A,B illustrates individual changes in all six outcomes displayed in relation to the foci of training with which the child started (hand use or mobility).

### 3.2. Is the Overall Outcome Influenced by Whether the Infants Train Mobility or Hand Use First?

The order in which the two different foci of training were performed had no significant effect on the changes in any outcome measure, nor at the GMFM-66 (*p* = 0.56); the scores on the locomotion (*p* = 0.44), stationary (*p* = 0.43), grasping (*p* = 0.71), or visuomotor (*p* = 0.62) subscales of the PDMS; or the HAI (*p* = 0.99). 

### 3.3. Was There any Difference in Improvement between the First and Second Period of Training?

Higher improvements after the first training period were seen in one outcome measures in both gross and fine motor assessments. This was seen in HAI for hand use and the stationary subscale in PDMS-2 for mobility (*p* = 0.0455 and 0.0055, respectively). For the other outcomes, the grasping and visuomotor subscales in the PDMS-2 (*p* = 0.531 and 0.345, respectively), as well as for GMFM-66 and the locomotion subscale in PDMS-2 (*p* = 0.1054 and 0.657, respectively), there were no effects of the order of the training period. 

### 3.4. Did the Children Get Better at What They Trained?

**Figure 3:**
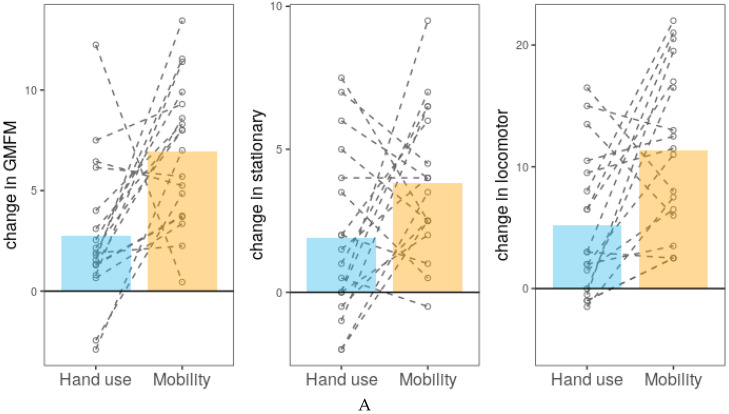

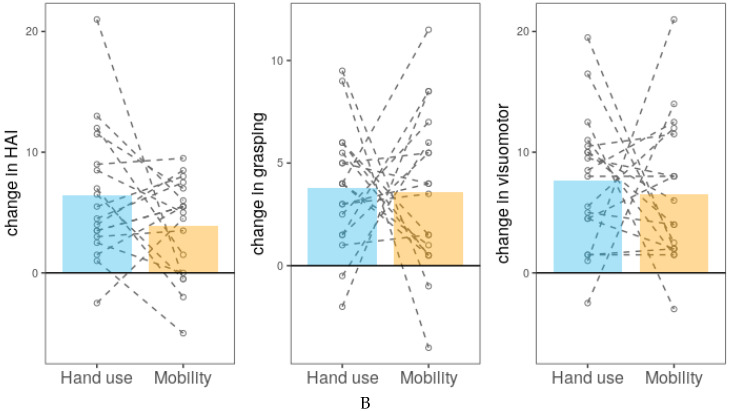
Average of group and individual changes (for both periods) in outcomes for hand use and mobility training. The changes in scores is calculated as a difference between scores after and before a training period. The bars represent averages across all subjects. The dots represent results for each individual averaged across 2 periods of hand training and 2 periods of mobility training connected with dashed lines. (**A**) Changes in gross-motor function. Left: Changes in the GMFM-66. Middle: Changes in the score on the stationary subscale of the PDMS-2. Right: Changes in the score on the locomotor subscale of the PDMS-2. (**B**) Changes in fine-motor function. Left: Changes in the HAI score. Middle: Changes in the score on the grasping subscale of the PDMS-2. Right: Changes in the score on the visuomotor subscale of the PDMS-2. The range of the different scales is described in the Methods section.

Combining the changes in scores for both time periods, we next examined whether gross-motor function improved to a greater extent after mobility training than during the training of hand use and vice versa. All outcomes related to gross motor function improved more after mobility than after hand use training (GMFM-66, *p* < 0.000001); and changes in the locomotion and stationary subscales of the PDMS-2 (*p* = 0.0027 and 0.039, respectively). In contrast, improvement in fine motor function outcome variables did not differ following the different types of training (HAI: *p* = 0.171; and the grasping and visuomotor subscales of the PDMS-2: *p* = 0.89 and 0.56, respectively) (Figure 3). We repeated the analyses controlling for AIMS at baseline, GMFCS—E&R level, cognition (BSID-III), and CP diagnosis at two years of age. The results remained qualitatively the same.

### 3.5. What Happened with Gross and Fine Motor Development during the Intermission of Motor Training?

To further characterize the improvements resulting from training, we compared the changes in outcomes following the periods of hand use or mobility training with potential changes following the period of intermission of motor training (T2–T3). As expected, the improvement in gross-motor function was significantly greater after mobility training in comparison to the period of intermission of motor training (GMFM-66: *p* < 0.00011; the locomotion and stationary subscales of the PDMS-2: *p* = 0.019 and 0.018, respectively). However, and in contrast, the improvements in fine-motor function did not differ following the training of hand use and the intermission of motor training (the HAI: *p* = 0.881; the grasping and visuomotor subscales of the PDMS-2: *p* = 0.59 and 0.21, respectively). Since the period of intermission with no motor training varied in length, we repeated the analyses, controlling for the potential influence of the HINE at baseline and the number of weeks involved in the study. The results remained qualitatively the same.

## 4. Discussion

This is one of the first studies describing early development, from a longitudinal perspective, during the first year of life for children with CP or other neurodevelopmental disorders. The design of the present study made it possible to study the outcome in relation to training type (mobility or hand use) and outcome in relation to the timing of the different intervention periods. All outcomes improved during the intervention period, which started when the children were 4–9 months old. Our hypothesis that even young children learn what they practice was confirmed with respect to gross motor function, which was most improved by the mobility training periods. The first period of training gave better results than the second training period at three-to-four months later for both hand use (on the HAI) and mobility training (on the stationary subscale of the PDMS-2,), but this was not seen in the other outcomes. However, the periodization of training, whether hand use or mobility was trained first, did not influence outcome at the end of the study. Importantly, this program had similar effects on the infants diagnosed with CP or other neurodevelopmental disorders, indicating that it is beneficial for all infants regardless of their future diagnosis. 

### 4.1. You Learn What You Practice 

Our hypothesis was that infants and young children, as in the cases of older children and adults, would learn what they practice, i.e., more improvements would be seen in the distinct foci of each training period when compared to the foci that was not currently addressed [13,16]. Our findings suggest that the same rules regarding specificity in outcome holds true across ages, since the highest rate of gross motor development was related to the time periods of mobility training. Interestingly, children continued to develop in gross motor function during the time periods of hand use training and during the intermission of no motor training (but at a slower rate), indicating that focused mobility training during a limited period might also have a longitudinal effect on development in very young children. These findings also strengthen the rationale for the training model; while training other functions, the children did not lose the useful and meaningful skills they had acquired from earlier training. Thus, our results appear to refute the common concern that a period of no physiotherapy might have a detrimental impact on the development of gross motor function. 

One possible explanation for the continued development of gross motor function even during periods without mobility training here could be that the training approach in this intervention was based on solution and coaching strategies [12,30,31]. We encouraged the creative exploration of the family´s competence and self-made decisions concerning everyday activities. The organization of the training program, as well as the interaction with professional therapists competent in specific areas, was much appreciated by the families. The parents reported that each type of training provided them with different skills of importance to their child [11]. The setting of goals together with therapists gave the parents a growing sense of competence, enabling them to plan ahead for the next step in their child´s development. Similar developments of parental skills and confidence have also been observed in previous studies involving families with older children [32,33,34].

By two years of age, half of our participants had received a diagnosis of CP and the other half had been diagnosed with a variety of other neurodevelopmental disorders. Though our study population was relatively small, we found no difference in outcome for these infants. The findings support the idea that the treatment concept is not diagnosis-specific, rather it is tailored to support the needs of each individual based on individualized, goal-directed, and self-initiated movements [12]. 

### 4.2. The Importance of Early Training 

The design of the present study allowed for the comparison of earlier or later interventions, with about four-to-five months between the first and second training period. This comparison revealed more extensive improvement in both mobility and hand use during the first training period. This finding is consistent with an earlier report on the effect of constraint-induced movement therapy (CIMT) on infants with unilateral CP [35]. This more pronounced improvement with earlier training is in agreement with well-established knowledge concerning the extensive activity-dependent plasticity and rapid development of the central nervous system during early life [4,36]. Another factor might have been that after parents, at the start of the study, were shown how to use activities and adjust these to their child’s ability and interests that boosts the first period of training for the first time, they could provide an environment that encouraged the child to practice, learn, and explore his/her own potential. There might also have been a saturation in training effect during the second period when the child´s potential came close to ceiling effect and the improvement went slower. Though the long-term effects of early intervention remain unclear, developing abilities at an early age allows skills to be practiced/repeated for a longer period during the most sensitive time of development. Theories on motor learning emphasize that repetition is one of the most important means of improvement [13].

### 4.3. Differences in the Outcomes for Mobility and Hand Use

Even though mobility and hand use are considered in similar ways by current theories of motor learning, our results here were less clear in the case of hand use. However, cognitive strategies are particularly important in connection with learning hand skills [37], and, moreover, the neural networks that control fine and gross motor functions differ. Acquiring new gross motor skills can be discussed in terms of implicit motor learning (primarily dependent on control of posture and coordination), whereas the development of fine motor functions involves more explicit learning (mainly of complex skills and procedures) and is more dependent on working memory [38]. Likewise, according to the literature of mirror neurons, repeated observations of actions play an important role in learning fine motor skills, and the link between grasp and language has previously been demonstrated in animal studies [39]. This suggests that even during the communication and mobility periods, the learning of fine motor skills might have been strengthening. An important factor is that the development of hand use is driven by the infant’s interest in exploring objects and thus closely linked to cognitive development. This challenge may render the parents´ role more demanding and perhaps less concrete. To complicate matters further, hand use is also affected by additional impairments, such as visual problems. The differences observed here concerning the effects of training on gross and fine motor skills may indicate that our methods for improving hand use in infants needs to be further improved. Most methods currently available for improving hand use have been developed for children with unilateral CP, whereas effective gross motor training for children with all forms of CP is already well established. 

### 4.4. Limitations and Clinical Implications

Both limitations and implications for future studies could be considered when interpreting the findings from the present study. In light of the relatively small size of our present study population, our findings must be considered as preliminary and be seen as hypothesis-generating and a guide to be used in future studies in order to improve effectiveness Additionally, there is a need to continue the development of early intervention programs and assessment tools that are valid, reliable, and sensitive to the group of infants considered at risk of CP or other neurodevelopmental disorders [40,41]. Regarding the measurement of outcomes here, the HAI was developed for infants with unilateral CP, and more knowledge is needed on the psychometrics of the instrument in children at risk of other forms of CP. Moreover, the presently available information concerning the assessment of gross motor development in children as young as our subjects with CP with the GMFM-66 is limited. We are also aware that the PDMS-2 is a norm-referenced test designed to characterize deviation from typical development, rather than measure outcome following an early intervention. Clearly, few psychometrically sound assessments of outcome following an early intervention involving children at risk of developing CP or other neurodevelopmental disorders are yet available, especially in the case of children who are more severely affected and whose small improvements might be more difficult to evaluate properly.

## 5. Conclusions

We have been able to provide some evidence for the concept “you learn what you practice,” especially with regards to gross motor development. We have also provided novel insights into how the timing, content, and structure of early interventions influence the outcome in infants—questions frequently asked in relation to many training approaches for older children as well [42,43]. Our current findings provide support for the broad (different types of training), specific (individualized training and goals), and intensive approach with different intervals, with parents as training providers (coached by therapists) in the home environment. Though early intervention appears to be beneficial, the long-time effects are probably influenced by many factors that vary widely between children and remain to be explored in detail.

## Figures and Tables

**Table 1:** Baseline demographic characteristics, diagnosis, and classifications of the participants involved in this study (*n* = 19).

**Characteristic**	
Gestational age in weeks: mean (SD)	33 (6.5)
CA age at the time of inclusion (T0) in months: mean (SD) and min–max	6.3 (1.62) 4–9
CA age at the end of the intervention (T5): mean (SD) and min–max	16.7 (2.23) 13–21
Gender: male/female	12/7
**Risk factors for cerebral palsy**	
Extreme premature/preterm/term birth	6/5/8
AIMS (raw score) (T0)	18.0 (6.87)
HINE (T0), Neurological symptoms	49.0 (11.03)
Behavioral signs	13.8 (0.91)
Twins, *n* (families)	4 (3)
**Diagnoses**	
Ataxic CP	**1**
Dyskinetic CP	3
Bilateral Spastic CP	5
Unspecified CP	1
Autism	1
Other neurological disease	6
Slight delay/typical development	1/1
**Classifications**	
GMFCS—E&R, I/II/III/IV/V	5/7/3/2/2
Mini MACS I/II/III/IV/V	7/3/7/0/2
**Intervention**	
Weeks involved in the intervention (T0–T5), mean (SD)	43.5 (5.88)

Abbreviations: CA = Corrected age for children born before week 37: extreme premature at <29 weeks and preterm at 29–36 weeks; AIMS = Alberta Infant Motor Scale; HINE = Hammersmith Infant Neurological Examination; GMFCS—E&R = Gross Motor Function Classification System—Expanded and Revised; and Mini MACS = Manual Ability Classification system (up to 4 years of age), CP = Cerebral Palsy.

**Table 2:** Alterations (means and 25–75 percentile) in the scores on the Peabody Developmental Motor subscales^2ed^ (PDMS, raw score), Gross Motor Function Measure-66 (GMFM-66) and Hand Assessment for Infants (HAI) after the end of intervention (T5) in comparison to the baseline (T0).

Assessments	T0—Base Line	T5—End of Intervention	*p* Value
PDMS, Stat	21.9 (18.8–25.0)	35 (32.7–37.3)	*p* = 0.0001
PDMS, Loc	19.7(15.8–23.6)	58 (45.1–70.9)	*p* < 0.0001
PDMS, Gr	17.5 (13.3–21.7)	36 (32.6–39.4)	*p* = 0.0002
PDMS, Vm	20 (14.4–25.6)	59.6 (50.5–68.2)	*p* < 0.0001
GMFM-66	27.7 (24.8–30.5)	48.4 (43–54)	*p* < 0.0001
HAI, BoHs	31.5 (17–45)	46.7 (43–57)	*p* = 0.0002

Stat = stationary; Loc = locomotion; Gr = grasping; Vm = visuomotor integration. BoHs = both hand score.
